# The *n*-3 Polyunsaturated Fatty Acids Supplementation Improved the Cognitive Function in the Chinese Elderly with Mild Cognitive Impairment: A Double-Blind Randomized Controlled Trial

**DOI:** 10.3390/nu9010054

**Published:** 2017-01-10

**Authors:** Yacong Bo, Xueyuan Zhang, Youli Wang, Jie You, Han Cui, Yiwei Zhu, Wei Pang, Wei Liu, Yugang Jiang, Quanjun Lu

**Affiliations:** 1 Department of Nutrition and Food Hygiene, College of Public Health, Zhengzhou University, Zhengzhou 450001, China; boyacong@163.com (Y.B.); qiu_contribution@163.com (X.Z.); zzu_youjie@126.com (J.Y.); 1zhuxic@126.com (H.C.); yiwei0726@163.com (Y.Z.); 2 The Center for Disease Control and Prevention of China Pingmei Shenma Group, Pingdingshan 467000, China; 15188318679@139.com; 3 Department of Nutrition, Tianjin Institute of Health and Environment Medicine, Tianjin 300050, China; hnyl2001@126.com (W.P.); zhu412825@126.com (W.L.)

**Keywords:** *n*-3 polyunsaturated fatty acids, mild cognitive impairment, elderly, cognition, basic cognitive aptitude tests

## Abstract

Objective: Intake of *n*-3 polyunsaturated fatty acids (*n*-3 PUFAs) may protect against mild cognitive impairment (MCI). However, there is still a lack of the *n*-3 PUFAs intervention in the elderly with MCI in China. The aim of the present study was to investigate the effect of *n*-3 PUFA supplementation on cognitive function in the Chinese elderly with MCI. Methods: Eighty six MCI individuals aged 60 years or older were randomly assigned to receive either *n*-3 PUFAs (480 mg DHA and 720 mg EPA per day, *n* = 44) or placebo (olive oil, *n* = 42) capsules. The changes of cognitive functions were assessed using Basic Cognitive Aptitude Tests (BCAT). Results: The mean age of participants was 71 years old, and 59% of the participants were men. *n*-3 PUFA supplementation was associated with improved total BCAT scores, perceptual speed, space imagery efficiency, and working memory (*p* < 0.01), but not with mental arithmetic efficiency or recognition memory (*p* > 0.05). Subgroup analysis by sex showed that *n*-3 PUFAs significantly improved perceptual speed (*p* = 0.001), space imagery efficiency (*p* = 0.013), working memory (*p* = 0.018), and total BCAT scores (*p* = 0.000) in males. However, in females, the significant beneficial effects can only be observed in perceptual speed (*p* = 0.027), space imagery efficiency (*p* = 0.006), and total BCAT scores (*p* = 0.015)—not working memory (*p* = 0.113). Conclusion: *n*-3 PUFAs can improve cognitive function in people with MCI. Further studies with different fish oil dosages, longer intervention periods, and larger sample sizes should be investigated before definite recommendations can be made.

## 1. Introduction

Cognitive impairment is a prevalent condition among the elderly and its burden tends to increase in parallel with increasing life expectancy. No effective pharmacological treatment has been identified to date [1]. Mild cognitive impairment (MCI) is an intermediate stage in the continuum from normal aging to dementia. With the increase in the percentage of elderly people, the prevalence of MCI is rapidly increasing, and systematic studies have suggested that the prevalence of MCI ranges from 0.5% to 42% worldwide [2] and from 2.4% to 35.9% in China [3]. Moreover, an individual with MCI has a 10-fold increased risk of developing dementia compared to cognitively normal individuals [4]. MCI has become an important stage for early intervention of dementia.

The *n*-3 polyunsaturated fatty acids (*n*-3 PUFAs), particularly eicosapentaenoic acid (EPA) and docosahexaenoic acid (DHA), might play a protective role against age-related cognitive decline. Clinical trials with *n*-3 PUFAs in healthy older adults [5,6,7], MCI patients [8,9], and Alzheimer’s disease (AD) [10,11] have been conducted, and these results suggest that people with MCI are more likely to respond.

Inflammation is a characteristic of many neurodegenerative diseases, including AD [12]. It is hypothesized that early prevention or management of inflammation could prevent cognitive decline of MCI and delay the onset of AD. The *n*-3 PUFAs have anti-inflammatory and pro-resolving properties in the periphery [13]. It has been shown to potently modulate neuroinflammation by decreasing the production of eicosanoids from arachidonic acid [14], while EPA and DHA derivatives were involved in the resolution processes related to inflammation, and may actively shut off inflammatory reactions [15]. However, to our knowledge, studies exploring the effect of *n*-3 PUFA supplementation in the Chinese elderly with MCI are limited. Therefore, the purpose of the current study was to investigate the effect of *n*-3 PUFA supplementation on cognitive function in Chinese elderly with MCI.

## 2. Materials and Methods

### 2.1. Participants

This was a community-based 6-month, randomized, double-blind, placebo-controlled trial among Chinese elderly with MCI. Participants were recruited from four communities in China Pingmei Shenma Group (Pingdingshan, China). Eight hundred and twenty elderly adults (≥60 years old) without physical or mental illness who were able to communicate were screened between February 2014 and May 2014. All subjects were asked to sign informed consent before the study. The Research Ethics Committee of Zhengzhou University approved the study protocol and the informed consent.

**Figure 1:**
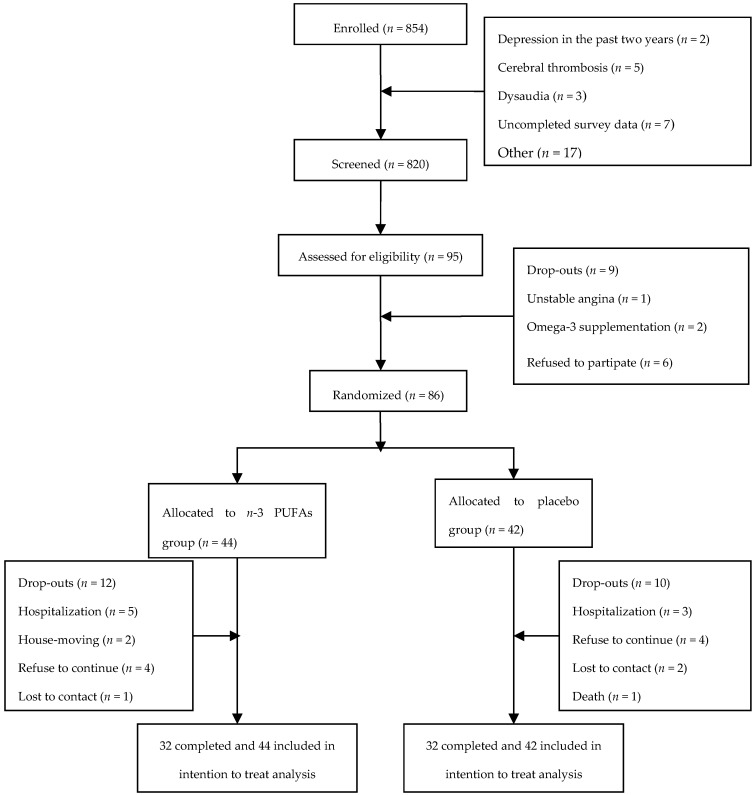
Flow of participants through the trial.

Face-to-face interviews were conducted by trained investigators using a Basic Information Questionnaire, a Minimum Mental State Examination (MMSE), an Activity of Daily Living (ADL) Scale and the Clinical Dementia Rating (CDR) to screen MCI. Individuals with probable MCI, according to the modified criteria of Petersen [16] were included in the current study. The inclusion criteria were as follows: (1) people with memory disorders or other cognitive disorders for at least 3 months; (2) an MMSE score of 24–26 for individuals who had more than 6 years of education, 20–22 for those who had less than 6 years, and 17–19 for participants without education; (3) having no disease that could affect our trail; and (4) not taking *n*-3 fatty acid supplements. Exclusion criteria were any type of newly diagnosed neurodegenerative disease, psychiatric disease, or mental disorder; taking *n*-3 preparations or vitamin supplements/drinks/injections with vitamin B6, folate, vitamin B12, vitamin E, or ginkgo in the past year; drug or alcohol abuse; or diabetes, cancer, or kidney failure. At last, 86 individuals with MCI were enrolled in the trial (Figure 1).

### 2.2. Randomization and Intervention

The randomization sequence was computer-generated by a blinded statistician not involved in data collection or analysis according to age and gender. Participants were randomized to receive either four 1 g soft gelatine capsules every nine days, where each capsule contained 120 mg of DHA and 180 mg of EPA (Royal DSM Company of Holland, Shanghai, China), and the total dosage was 480 mg of DHA and 720 mg of EPA daily, which was decided on the basis of previous fish oil trials [5,6], or an isocaloric placebo olive oil (each containing 550 mg of oleic acid). All participants were asked not to change their current dietary habits in the course of the study.

The study was approved by the Ethics Committee of the Zhengzhou University, China, and was in accordance with the declaration of Helsinki. All subjects gave written informed consent before participating the study. This study is ChiCTR-TRC-14004625 in the Chinese Clinical Trial Registry (http://www.chictr.org.cn).

### 2.3. Cognitive Function Test

Basic Cognitive Aptitude Tests (BCATs), the primary outcome, was used to evaluate the cognitive function of the subjects at baseline and Month 6. The BCAT, designed by the Institute of Psychology, Chinese Academy of Sciences, includes seven sub-items: digit copy, Chinese character comparison, mental arithmetic, Chinese character rotation, recall answer of mental arithmetic, recognition of two-word nouns, and recognition of meaningless figures. These seven sub-items were divided into five sections: perceptual speed (PS), mental arithmetic efficiency (MAE), space imagery efficiency (SIE), working memory (WM), and recognition memory (RM). The test lasted 10–30 min.

### 2.4. Blood Collection and Analysis

Blood samples were collected at baseline and after 6 months’ intervention. The fatty acid profile of plasma phosphatidylcholine was analyzed by gas chromatography. Total lipid was extracted from plasma using chloroform–methanol (2:1 *v*/*v*). Plasma phosphatidylcholine was isolated by solid phase extraction. Fatty acid methyl esters were prepared by incubation of purified plasma phosphatidylcholine with methanol in sulfuric acid and were separated using a Hewlett Packard 7890 gas chromatograph (Agilent, Stockport, Cheshire, UK) equipped with a 50 m × 0.33 μm × 0.2 mm BPX-70 fused silica capillary column (SGE Analytical Science, Trajan Scientific Europe Ltd., Crownhill, Milton Keynes, UK) and flame ionization detection. The concentrations of individual fatty acids in plasma phosphatidylcholine were determined by measurement of the peak area using ChemStation software (Agilent), and each fatty acid was expressed as a proportion of the total [17]. Calculations were based on the percentage contribution each fatty acid makes to the total fatty acids identified in plasma phosphatidylcholine. Cytokines (i.e., Interleukin-6 (IL-6), Interleukin-10 (IL-10), and tumor necrosis factor-α (TNF-α)) in plasma samples were measured by using a radioimmunoassay. Enzyme activity (i.e., cyclooxygenase (COX), lipoxygenase (LOX), and secretory phospholipase A2 (sPLA2)) of the samples was determined by double antibody-based sandwich enzyme-linked immuno-sorbent assay (ELISA). ELISA kit was purchased from Tsz Biosciences (San Francisco, CA, USA).

### 2.5. Data Analysis

Analyses were performed as intention-to-treat, defined as all participants randomized, regardless of whether they finished the full study protocol. The analysis of independent samples *t* tests for continuous variables and Chi square test for categorical variables were conducted to compare the baseline characteristics of the two groups. The independent samples *t* tests were also used to explore differences between the intervention and placebo groups. All analyses were carried out using SAS statistical software package (version 9.1; SAS Institute Inc., Cary, NC, USA). A two-sided *p* value < 0.05 was considered significant.

## 3. Results

### 3.1. Baseline Characteristics

A total of 854 subjects were screened for the study, and 86 subjects met the study criteria and were randomized to *n*-3 PUFA (44 subjects) or placebo (42 subjects). A flowchart of the study population is shown in Figure 1. Baseline characteristics (age, gender, education, and fatty acid composition) showed no significant differences between the two groups (Table 1).

### 3.2. Changes in Plasma Fatty Acids Composition

After the intervention, the proportions of DHA and EPA measured in the plasma of peripheral blood in the subjects of the intervention group were significantly higher than that of the subjects in the placebo group. The subjects had high compliance with *n*-3 PUFA capsule intake. No significant difference was found for the proportion of other PUFAs between these two groups (Table 2).

### 3.3. Cognitive Function Changes

Compared with pre-intervention, the total score of the BCAT test in the two groups were both improved. The intervention group had more significant improvement than the placebo group (*p* < 0.0001), which indicated that their cognitive function was improved in general. Perceptual speed, space imagery efficiency, and working memory of the subjects in the intervention group were significantly improved after the six-month intervention (*p* < 0.05). However, the change in mental arithmetic efficiency and recognition memory showed no difference between the two groups (*p* > 0.05) (Table 3).

**Figure 2:**
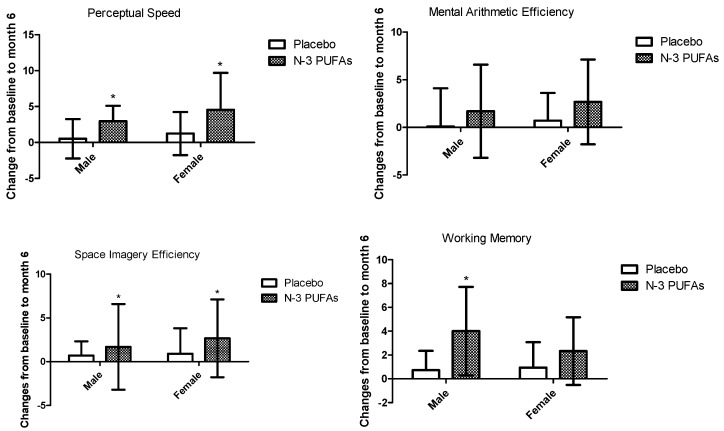

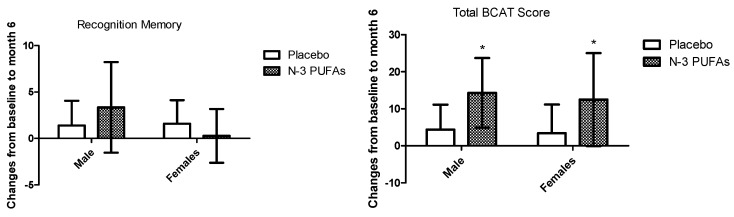
Changes in perceptual speed, mental arithmetic efficiency, space imagery efficiency, working memory, and recognition memory between *n*-3 PUFAs and control groups stratified by sex. Data represent mean changes over 6 months with SD. The *n*-3 PUFAs effect and significance level are based on independent samples *t* tests.

In order to explore whether there is some difference between males and females, the beneficial effects on cognitive function were analyzed in separate strata of sex. Compared with the placebo group, *n*-3 PUFAs significantly improved perceptual speed (*p* = 0.001), space imagery efficiency (*p* = 0.013), working memory (*p* = 0.018), and total BCAT scores (*p* < 0.001) in males. However, significant beneficial effects can only be observed in perceptual speed (*p* = 0.027), space imagery efficiency (*p* = 0.006), and total BCAT scores (*p* = 0.015) in females (Figure 2).

### 3.4. Changes in the Plasma Indicators Levels

The *n*-3 PUFA supplementation led to a significant decrease in IL-6, TNF-α levels, and sPLA2 activity (*p* < 0.05). However, no significant changes in these parameters before and after the test in the placebo group were observed (Table 4).

## 4. Discussion

This randomized clinical trial demonstrated that six months of supplementation with 480 mg/day DHA and 720 mg/day EPA could improve perceptual speed, space imagery efficiency, and working memory in older adults with MCI.

There is still no consensus on the *n*-3 PUFA dose. EFSA and FAO recommended that the acceptable macronutrient distribution range (AMDR) of EPD + DHA is 0.25–2 g/day for the elderly [18,19]. In addition, Geleijnse et al. provided an EPA/DHA ratio of 3:2 [20]. In the current study, we provide 480 mg of DHA and 720 mg of EPA (ratio of 3:2) daily for each participant in the intervention group.

As described in reviews that consider various tools used for cognitive testing, MMSE has been widely used by researchers in MCI screening because of its easy operation and less time consuming. MMSE may be a relatively crude screening tool, while the BCAT score could sensitively reflect a detailed change in cognitive function [21]. Cognitive functions of the two groups (*n*-3 PUFAs and placebo) were evaluated by the BCAT software (Institute of Psychology, the Chinese academy of Social Sciences, Beijing, China) [22] before and after the supplementation. Daily oral *n*-3 PUFA supplementation for six months in subjects with MCI beneficially affected perceptual speed, space imagery efficiency, and working memory, which were in agreement with other trials reporting a protective effect of *n*-3 PUFA supplementation on cognitive function in patients with MCI [8,9,23]. Intriguingly, working memory and total score of the BCAT test in these two groups were both improved compared with pre-intervention. It is speculated that the test score may be affected by proficiency of the subjects.

The findings from this randomized, double-blind, placebo-controlled study found that *n*-3 PUFA supplements in individuals with MCI might benefit in cognition, which supports previous intervention studies [24,25,26,27]. These findings support other research evidence that indicates that *n*-3 PUFA supplementation improves cognition in individuals with MCI or very mild AD [10,24,28]. However, the findings from this study contradict some research evidence showing that this type of intervention does not benefit individuals with mild to moderate AD [29,30,31]. The equivocal evidences of *n*-3 PUFA supplements on participants with MCI might be ascribed to the relatively small sample sizes. Therefore, future research with larger samples sizes and longer study durations are warranted to clarify this effect.

Several studies have shown that DHA and EPA can exert anti-inflammatory activities, and their beneficial effects are commonly ascribed to this property [32,33]. IL-6, IL-10, and TNF-α are common cytokines. A large number of studies have shown that *n*-3 PUFA intervention can reduce human plasma IL-6, IL-10, TNF-α, and secretory phospholipase A2 (sPLA2) levels but increase plasma IL-10 levels to achieve anti-inflammatory effects [34,35,36,37], but the reports have been inconsistent in MCI subjects [38]. The current results show a significant decrease in IL-6, TNF-α, and sPLA2 after *n*-3 PUFA supplementation.

The results of this study suggest that *n*-3 PUFA supplementation may have positive benefits in individuals with MCI and may also be beneficial in primary prevention in people with MCI. Several potential mechanisms might explain the positive effect of *n*-3 PUFAs. First, *n*-3 PUFAs constitute more than 30% of the membrane phospholipid composition, regulating membrane structure, fluidity, and signal-transduction [39]. In addition, *n*-3 PUFAs modulate gene expression patterns that facilitate BDNF-mediated synaptic plasticity [40], influence B-vitamin or homocysteine pathways [41], and activate energy-generating mechanisms involved in glucose and lipid metabolism [42]. Moreover, *n*-3 PUFAs may protect cognitive function by modulating the immune response to amyloid-β [43].

Several potential limitations of this study should also be acknowledged. First, the sample size was relatively small. However, we believe that the findings from this study still contribute to the body of evidence that helps to determine the benefits of *n*-3 PUFAs in cognition in MCI individuals. Another limitation was the high dropout rate (25%), which could bias the results via intention-to-treat analysis. The dropout rate was similar in both groups, and baseline characteristics of the dropouts were comparable to those participants included in the final analysis.

## 5. Conclusions

In summary, this study confirmed the protective effect of *n*-3 PUFAs on the cognitive function of the elderly with MCI. Large, high-quality randomized controlled trials with elderly MCI patients are needed to further explore potential mechanisms and the effective intervention dosage.

## Figures and Tables

**Table 1:** Baseline characteristics of subjects ^1^.

Variable	Placebo (*n* = 42)	*n*-3 PUFAs (*n* = 44)	*t*/*X*^2^	*p*
Age (year)	70.45 ± 6.82 ^2^	71.75 ± 5.68	0.961	0.339 ^3^
Gender			0.002	0.967 ^4^
Male	25	26		
Female	17	18		
Education level			1.038	0.595 ^4^
Illiterate	1	1		
Primary school	11	16		
Junior high school and above	30	27		
EPA (%)	0.96 ± 0.59	0.80 ± 0.16	−1.728	0.088 ^3^
DHA (%)	1.62 ± 0.43	1.55 ± 0.39	−0.698	0.487 ^3^
AA (%)	0.14 ± 0.04	0.15 ± 0.03	0.875	0.384 ^3^
ALA (%)	0.63 ± 0.17	0.68 ± 0.19	−1.286	0.202 ^3^
LA (%)	22.39 ± 3.26	22.34 ± 2.86	−0.075	0.940 ^3^
BCAT	33.17 ± 15.01	31.16 ± 15.41	−0.612	0.542 ^3^
MMSE	25.62 ± 1.68	25.11 ± 1.66	−1.403	0.164 ^3^

^1^ The fatty acids composition was expressed as a relative percentage of the total amount of fatty acids reported; ^2^ Mean ± standard deviation (SD); ^3^ Unpaired *t*-test; ^4^ Chi-square test. EPA: eicosapentaenoic acid; DHA: docosahexenoic acid; AA: arachidonic acid; ALA: alpha linolenic acid; LA: linoleic acid, MMSE: Mini-mental State Examinatlon; BCAT: Basic Cognitive Aptitude Tests.

**Table 2:** Changes in plasma PUFA levels of MCI patients ^1^.

	Baseline	After Treatment	Difference	*p*
EPA (%)				
Placebo (*n* = 42)	0.96 ± 0.59	0.92 ± 0.58 ^2^	−0.05 ± 0.19	<0.0001 ^3^
*n*-3 PUFAs (*n* = 44)	0.80 ± 0.16	1.16 ± 0.56	0.36 ± 0.52	
DHA (%)				
Placebo (*n* = 42)	1.62 ± 0.43	1.36 ± 0.42 ^2^	−0.25 ± 0.43	<0.0001 ^3^
*n*-3 PUFAs (*n* = 44)	1.55 ± 0.39	1.89 ± 0.56 ^2^	0.34 ± 0.42	
AA (%)				
Placebo (*n* = 42)	0.14 ± 0.04	0.15 ± 0.05	−0.01 ± 0.05	0.066
*n*-3 PUFAs (*n* = 44)	0.15 ± 0.03	0.14 ± 0.04	0.01 ± 0.04	
ALA (%)				
Placebo (*n* = 42)	0.63 ± 0.17	0.65 ± 0.15	0.003 ± 0.12	0.582
*n*-3 PUFAs (*n* = 44)	0.68 ± 0.19	0.68 ± 0.19	0.02 ± 0.20	
LA (%)				
Placebo (*n* = 42)	22.39 ± 3.26	22.22 ± 3.02	−0.17 ± 2.48	0.333
*n*-3 PUFAs (*n* = 44)	22.34 ± 2.86	21.67 ± 2.83	−0.66 ± 2.15	

^1^ Data expressed as mean ± SD; ^2^ Significantly different from baseline, *p* < 0.05; ^3^ Significantly different from placebo group, *p* < 0.05.

**Table 3:** The effect of *n*-3 PUFAs on the BCAT scores of MCI patients ^1^.

Outcome Measure	Overall BCAT Score			*p*
	Baseline	After Treatment	Difference	
PS				
Placebo (*n* = 42)	5.62 ± 3.84	6.43 ± 4.62	0.81 ± 2.83	0.0002 ^3^
*n*-3 PUFAs (*n* = 44)	5.22 ± 4.40	8.84 ± 4.65 ^2^	3.61 ± 3.69	
MAE				
Placebo (*n* = 42)	7.90 ± 5.33	8.70 ± 6.02	0.33 ± 3.58	0.055
*n*-3 PUFAs (*n* = 44)	7.09 ± 4.83	8.63 ± 4.60	2.09 ± 4.68	
SIE				
Placebo (*n* = 42)	6.29 ± 3.38	6.28 ± 2.48	0.00 ± 3.22	0.0002 ^3^
*n*-3 PUFAs (*n* = 44)	4.46 ± 2.34	6.81 ± 3.23 ^2^	2.45 ± 2.72	
WM				
Placebo (*n* = 42)	5.47 ± 3.80	6.85 ± 4.80	1.38 ± 2.66	0.0047 ^3^
*n*-3 PUFAs (*n* = 44)	5.32 ± 2.92	8.63 ± 4.06 ^2^	3.32 ± 3.45	
RM				
Placebo (*n* = 42)	7.52 ± 4.85	9.50 ± 5.16	1.98 ± 3.13	0.579
*n*-3 PUFAs (*n* = 44)	9.32 ± 7.69	10.86 ± 6.71	1.55 ± 3.96	
Total				
Placebo (*n* = 42)	33.17 ± 15.01	37.17 ± 16.85	4.00 ± 7.07	<0.0001 ^3^
*n*-3 PUFAs (*n* = 44)	31.16 ± 15.41	44.73 ± 13.87 ^2^	13.57 ± 10.72	

^1^ PS: perceptual speed; MAE: mental arithmetic efficiency; SIE: space imagery efficiency; WM: working memory; RM: recognition memory; ^2^ Significantly different from baseline, *p* < 0.05; ^3^ Significantly different from placebo group, *p* < 0.05.

**Table 4:** The effect of *n*-3 PUFAs on the plasma indicators levels of MCI patients ^1^.

	Baseline	After Treatment	Difference	*p*
IL-6, pg/mL				
Placebo (*n* = 42)	120.90 ± 41.60	109.26 ± 37.21	−11.64 ± 42.28	0.018 ^3^
*n*-3 PUFAs (*n* = 44)	120.60 ± 52.64	85.66 ± 53.01 ^2^	−34.94 ± 46.18	
IL-10, ng/mL				
Placebo (*n* = 42)	166.10 ± 70.54	152.43 ± 43.43	−13.67 ± 67.74	0.649
*n*-3 PUFAs (*n* = 44)	136.42 ± 48.96	128.69 ± 37.53	−7.73 ± 71.22	
TNF-α, fmol/mL				
Placebo (*n* = 42)	17.17 ± 7.98	15.43 ± 5.01	−1.74 ± 8.15	0.027 ^3^
*n*-3 PUFAs (*n* = 44)	19.00 ± 9.89	13.09 ± 7.86 ^2^	−5.91 ± 9.03	
COX, U/L				
Placebo (*n* = 42)	545.32 ± 146.12	539.92 ± 152.23	−5.40 ± 169.98	0.259
*n*-3 PUFAs (*n* = 44)	543.34 ± 146.81	579.94 ± 125.21	36.59 ± 172.64	
LOX, IU/L				
Placebo (*n* = 42)	128.83 ± 25.95	131.87 ± 21.10	3.04 ± 23.47	0.558
*n*-3 PUFAs (*n* = 44)	118.30 ± 26.14	124.61 ± 24.27	6.31 ± 27.63	
sPLA2, ng/L				
Placebo (*n* = 42)	944.00 ± 201.36	940.47 ± 233.43	−3.53 ± 267.60	0.052 ^3^
*n*-3 PUFAs (*n* = 44)	1002.55 ± 204.43	888.97 ± 211.82 ^2^	−113.58 ± 249.81	

^1^ Data expressed as mean ± SD; ^2^ Significantly different from baseline, *p* < 0.05; ^3^ Significantly different from placebo group, *p* < 0.05. IL-6: interleukin-6; IL-10: interleukin-10; TNF-α: tumor necrosis factor-alpha; COX: cyclooxygenase; LOX: lipoxygenase; sPLA2: secretory phospholipase A2.
